# Kinetics of calcium binding to dental biofilm bacteria

**DOI:** 10.1371/journal.pone.0191284

**Published:** 2018-01-31

**Authors:** Tarcísio Jorge Leitão, Jaime Aparecido Cury, Livia Maria Andaló Tenuta

**Affiliations:** 1 Department of Physiological Sciences, Piracicaba Dental School, University of Campinas, Piracicaba, São Paulo, Brazil; 2 Department of Dentistry II, Federal University of Maranhão, São Luis, Maranhão, Brazil; 3 Department of Cariology, Restorative Sciences and Endodontics, School of Dentistry, University of Michigan, Ann Arbor, MI, United States of America; National Institute of Technology Rourkela, INDIA

## Abstract

Dental biofilm bacteria can bind calcium ions and release them during a pH drop, which could decrease the driving force for dental demineralization (i.e. hydroxyapatite dissolution) occurring at reduced pHs. However, the kinetics of this binding and release is not completely understood. Here we validated a method to evaluate the kinetics of calcium binding and release to/from *Streptococcus mutans*, and estimated the importance of this reservoir as a source of ions. The kinetics of calcium binding was assessed by measuring the amount of bound calcium in *S*. *mutans* Ingbrit 1600 pellets treated with PIPES buffer, pH 7.0, containing 1 or 10 mM Ca; for the release kinetics, bacterial pellets previously treated with 1 mM or 10 mM Ca were exposed to the calcium-free or 1 mM Ca PIPES buffer, pH 7.0, for up to 60 min. Binding and release curves were constructed and parameters of kinetics were calculated. Also, calcium release was assessed by exposing pellets previously treated with calcium to a pH 5.0 buffer for 10 min. Calcium binding to bacteria was concentration-dependent and rapid, with maximum binding reached at 5 min. On the other hand, calcium release was slower, and according to the calculations, would never be complete in the groups pretreated with 10 mM Ca. Decreasing pH from 7.0 to 5.0 caused a release of calcium able to increase the surrounding fluid calcium concentration in 2 mM. The results suggest that dental biofilm bacteria may act as a calcium reservoir, rapidly binding ions from surrounding fluids, releasing them slowly at neutral pH and promptly during a pH drop.

## Introduction

Bacteria have a considerable capacity to bind calcium ions, which has implications in many processes, such as signaling, the transport of sugars and proteins, aggregation and formation of biofilms [1,2,3]. Specifically in dental biofilms, this capacity could have a significant role in the dynamics of the dental caries, since calcium, being on the ions of hydroxyapatite from the tooth mineral, if released from the bacteria to the fluid phase of the biofilm, could reduce the driving force for dental demineralization [4,5,6]. Moreover, calcium may function as a bridge between the anionic groups present in the bacterial surface and fluoride [7], and fluoride has an substantial effect on dental caries, reducing dental demineralization and enhancing dental remineralization [8,9]. In this respect, the formulation of innovative therapies to reduce the demineralization process, based on calcium enrichment of dental biofilm [10,11], may rely upon an understanding of the kinetics of binding and release of these ions to dental biofilm bacteria.

There are few studies that evaluated the kinetics [12] and capacity [5] of calcium binding/release from dental bacteria as a function of time and pH. Rose et al., in a series of studies [2, 5, 7, 13], attempted to estimate the importance of biofilm bacteria as reservoir of ions to interfere in dental demineralization. However, in these studies, calcium binding was estimated by the decrease in calcium concentration in solutions put in contact with the tested bacteria. This is not ideal, since the rate and capacity to bind and release calcium is a function of its concentration in the medium [14,15], which was used as the response variable in these studies. In addition, the potential release of calcium during a pH drop was only estimated by the differential binding of calcium at distinct pHs [5,7], but not by directly measuring calcium release under such conditions.

Also, in a more recent attempt to study the calcium (and fluoride) binding capacity to dental biofilm bacteria [16], the high concentrations used allow the formation, among the bacterial cells, of precipitated mineral, such as calcium fluoride; this may provide an unreliable estimation of bacterial calcium (and fluoride) binding and its effect on bacterial metabolism.

Thus, to estimate the kinetics of calcium binding and release from dental biofilm bacteria while avoiding methodology artifacts, we validated a new method to assess the amount of calcium bound to dental biofilm bacteria and used it to evaluate the kinetics of calcium binding and release, as well as to estimate the release of bound calcium to the surrounding fluid during a pH drop.

## Materials and methods

### Bacterial preparation

**Planktonic cells of the** *S*. *mutans* Ingbritt 1600 **were** cultivated in Tryptone-yeast extract broth (TYB) (Difco Labs., Detroit, USA) supplemented with 0.25% glucose for 18 h at 10% CO_2_ and 37°C. Bacterial pellets were separated by centrifugation and sequentially washed using sonication (Vibra Cell sonicator, Sonics and Materials, Danbury, USA), at 7 W for 1 min, to remove remnants of culture broth and unbound Ca, first in 0.05 M PIPES buffer, pH 7.0, followed by 0.01 M EDTA solution, and again in PIPES buffer. Between each washing, the pellet was recovered by centrifugation. After this procedure, the pellets were re-suspended in 0.05 M PIPES buffer and aliquots were transferred to pre-weighted 1.5-mL microcentrifuge tubes. These tubes were centrifuged (21,000 *g*, 5 min, 4°C) and the supernatant carefully discarded under microscope using a vacuum pump (Gilson Aspiration Station F110741, Middleton, WI, USA). Lastly, the bacterial pellets were weighed (± 0.01 mg) for calculation of the amount of calcium treatment solution to be added.

### Protocol validation

Details of this step can be found at http://dx.doi.org/10.17504/protocols.io.kuqcwvw. Briefly, *Streptococcus mutans* IB1600 pellets (from approximately 80–120 mg) were harvested by centrifugation in 1.5-mL microcentrifuge tubes. To these pellets, 0.05 M PIPES (piperazine-N, N’-bis [2-ethanesulphonate]; Sigma Biochemicals) buffer containing 1 or 10 mM Ca^++^ (CaCl_2_), pH 7.0, at 37°C, was added and the tubes were vortexed (AP 56, Phoenix, Araraquara, SP, Brazil) for 30 s. Tubes were maintained at 37°C for 60 min to allow complete binding. The proportion of treatment solution/bacteria was 15 μL/mg bacteria for the 1 mM Ca solution and 7.5 μL/mg bacteria for the 10 mM Ca solution. After 60 min, the bacteria were separated from the treatment solution by centrifugation (21,000 *g*, 5 min, 4°C), and supernatant was collected for determination of calcium concentration; the bacterial pellet was again centrifuged and the remaining treatment solution was carefully vacuum-aspirated with a micropipette under microscope using a vacuum pump (Gilson Aspiration Station F110741, Middleton, WI, USA). Bound calcium was extracted and determined after acid extraction as described below.

Four independent experiments with triplicate samples in each repetition were conducted.

### Calcium binding kinetics

Calcium binding and release kinetics experiments are described in details at http://dx.doi.org/10.17504/protocols.io.mamc2c6. *S*. *mutans* pellets were exposed to 0.05 mM PIPES buffer pH 7.0, containing 1 mM Ca or 10 mM Ca, at 37°C, for 5, 10, 30 or 60 min, at a proportion of 150 μL/mg of bacteria. A control group was treated with PIPES buffer without calcium for 60 min. Additionally, pellets pretreated with 1 mM Ca for 10 min were re-equilibrated with a solution containing 10 mM Ca for 60 min.

After the specified equilibrium time, the bacteria were separated from the test solution by centrifugation (21,000 *g*, 5 min, 4°C), and bound calcium was extracted and determined as described below.

Three independent experiments, with duplicate samples in each, were conducted (n = 3).

### Calcium release kinetics

Pellets pretreated with 1 or 10 mM Ca PIPES buffer for 10 min were re-equilibrated with calcium-free PIPES buffer at 37°C, for 10, 30 or 60 min, at proportion **of** 150 μL/mg of bacteria. Additionally, pellets pretreated with solution containing 10 mM Ca were equilibrated with PIPES buffer containing 1 mM Ca. A control group in which the bacteria were pretreated and re-equilibrated with PIPES buffer without calcium was included.

After 10, 30 and 60 min, the bacterial suspension was centrifuged; the supernatant was collected for determination of calcium concentration to ensure that there was no increase of concentration in treatment solution by calcium release from bacteria; the remaining bound calcium was acid-extracted from the bacterial pellet and the calcium concentration determined.

Release kinetics curves were constructed and parameters of release kinetics were calculated for three experiments (n = 3) with duplicate samples in each repetition. Calcium concentration found in bacterial pellets after pretreatment with 1 or 10 mM were plotted at time zero.

### Calcium release as a function of pH

Details of this experiment are available at http://dx.doi.org/10.17504/protocols.io.mapc2dn. Bacterial pellets pretreated with 1 or 10 mM Ca, 0.05 M PIPES buffer, pH 7.0, for 10 min, were treated with 0.05 M PIPES (pH 7.0, negative control), 0.5 M acetate buffer (pH 5.0, experimental group) or 0.5 M HCl (pH 1.86, positive control) at a proportion of 30% of the treatment solution per bacterial weight (similar to the proportion of fluid/solids in *in vivo* plaque [17]), at 37°C, for 10 min. The distinct pH solutions contained the same calcium concentration as the pretreatment fluid to avoid the effect of low calcium concentration on the phenomenon of release. An increase in final calcium concentration in the treatment solution was considered indicative of calcium release from the bacterial pellet as a function of pH.

### Extraction and determination of the bound calcium

In all experiment, bound calcium was extracted from the bacterial pellets by treatment with 0.5 M HCl (10 μL/mg bacterial wet weight) for 3 h at room temperature under constant agitation [18, 19]. The acid extract was collected after centrifugation and used for calcium determination.

Calcium was determined using the Arsenazo III colorimetric reagent [20], at 650 nm, in 96-well microplates, using a microplate reader (Multiskan Spectrum, Thermo Scientific, Vanta, Finland). All acid samples were neutralized using an equivalent concentration of NaOH. Calcium standards were prepared from CaCl_2_, at the same background composition as the samples.

### Data analysis

In protocol validation, the calcium amounts removed from the treatment solution and bound to bacteria were compared by the *t*-test.

For kinetics experiments, binding kinetics curves were fitted and affinity parameters were calculated (n = 3 experiments with samples duplicate at each repetition).

For the binding experiment, the equilibrium binding constant (K_d_) and maximum binding capacity (Ca_bmax_) were estimated using the equation: [Ca_b_] = Ca_bmax_*{1 – exp^[-Kon*([Caf]+Kd) *t]}^, where, [Ca_b_] = bound calcium; K_on_ = association rate constant; [Ca_f_] = concentration of treatment solution; and t = time in minutes. For the calcium release experiment, the calcium retained in release plateau (Ca_b∞_) and half-life values were estimated by: [Ca_b_] = (Cab0—Ca_b∞_)*exp^(-Koff *t)^ + Ca_b∞_, where, Ca_b0_ = initial bound calcium and K_off_ = rate of dissociation. Data were analyzed by GraphPad Prism 6.0 (GraphPad Prism Software Inc, CA, USA).

In the experiment of calcium release as a function of pH, initial and final calcium concentrations within each pH were compared by paired t-test. The amount of calcium released at each pH, according to the pretreatment condition (1 or 10 mM) was compared by t-test.

The normality of error distribution and the homogeneity of variance were checked for each response variable and these assumptions were satisfied. The SAS system (SAS Software, version 9.0; SAS Institute Inc., Cary, NC, USA) was used in the analysis, and the significance level was set at 5%.

## Results

*Streptococcus mutans* was used here because it is the major caries-related species, and also represents the dominant streptococci genus in the dental biofilm [21].

### Protocol validation

Unlike previous studies, we aimed to determine the amount of bound calcium to bacteria by direct extraction using strong acid, a method previously employed with this purpose [6, 11, 14, 18, 19]. To ensure that the method was capable of detecting bacteria-bound calcium, we designed a validation experiment. Our methods would be valid if the amount of calcium extracted from bacteria pellet was similar to the amount of calcium reduced from the treatment solution.

In order to allow a reliable estimation of the amount of calcium decrease from the treatment solution, we adjusted the volume of the treatment solutions: 15 μL of solution/mg of bacterial pellet was used for the 1 mM Ca treatment and 7.5 μL of solution/mg of bacterial pellet for the 10 mM Ca treatment. Considering that the 10 mM Ca treatment would have 10 times higher amount of Ca than the 1 mM Ca treatment at a given volume, we reduced the volume of solution in the 10 mM Ca treatment to be able to detect a decrease higher than 25% in the Ca concentration in the treatment solution.

The results confirmed that the amount of calcium bound to bacteria and the amount calcium removed from the treatment solution was not significantly different (paired-t test, p>0.05) (Table 1).

**Table 1:** Calcium (Ca) removed from the treatment solution and bound to bacterial pellet (mean ± sd; n = 4) after 60-min equilibrium with 1 or 10 mM Ca treatments.

Ca concentration	Ca removed from the solution (μmol)	Ca bound to bacterial pellet (μmol)
**1 mM**	**0.29** ± 0.08	**0.28** ± 0.09
**10 mM**	**1.90** ± 0.41	**1.83** ± 0.26

### Binding kinetics

In the binding kinetics experiments, the proportion of treatment solution to bacteria (150 μL/mg wet weight) was high enough to avoid a significant change in calcium concentration in the solution due to the binding.

**Fig 1:**
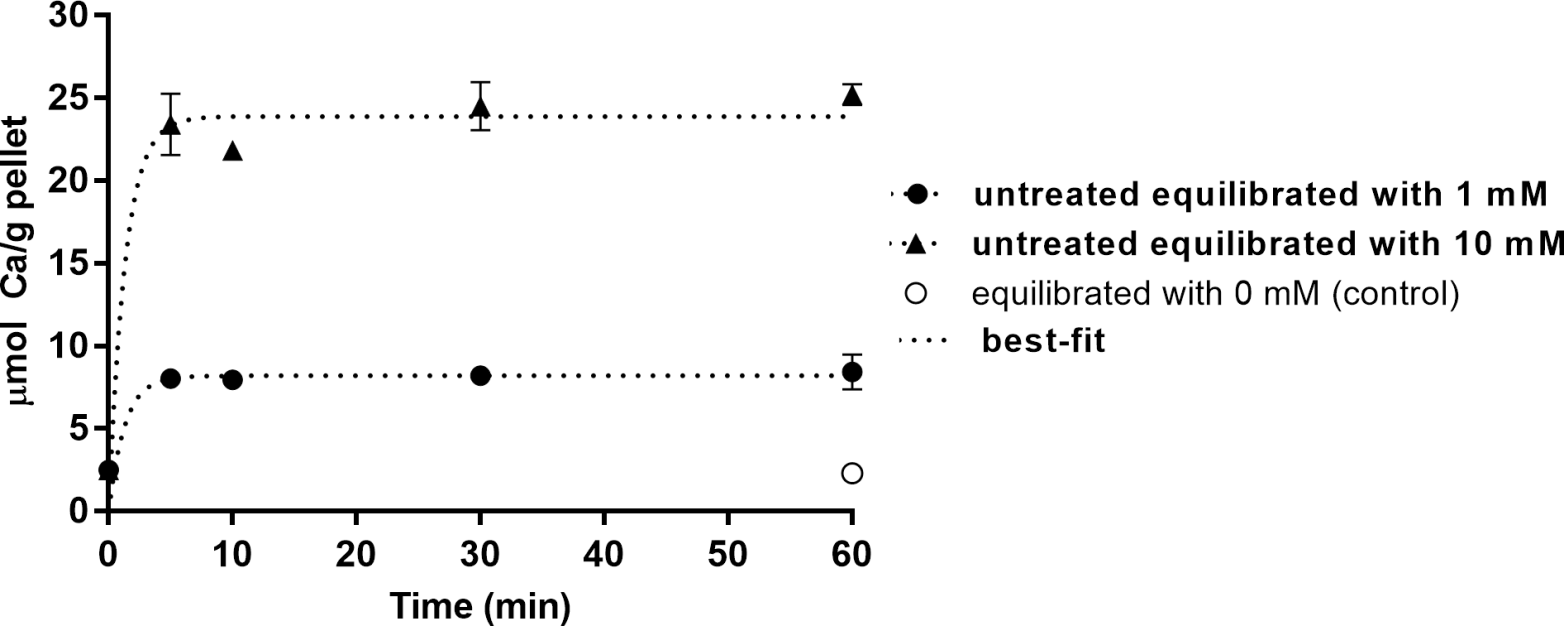
Kinetics of calcium binding to *S*. *mutans*. Ca concentration in the bacterial pellet as a function of time and of Ca concentration in the treatment solution (mean ± SD, n = 3).

Fig 1 shows the kinetics of calcium binding to bacteria at 1 or 10 mM. These concentrations were chosen to represent, respectively, the resting calcium biofilm fluid concentration [19] and a 10 times higher calcium concentration which can be found in biofilm fluid after a sugar challenge [4]. More than 90% of binding occurred in the first 5 min.

**Fig 2:**
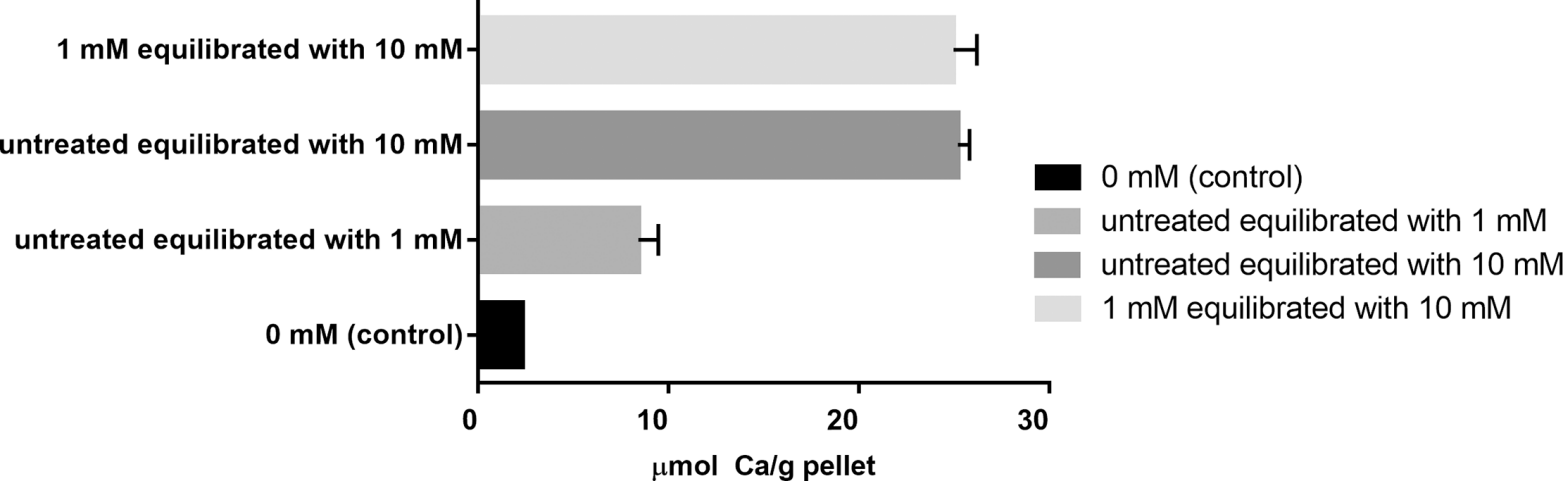
Calcium binding at equilibrium. Calcium concentration (μmol/g, mean ± sd, n = 3) in the bacterial pellet after 60 min of equilibrium of bacteria with PIPES buffer pH 7.0, containing 0 mM, 1 mM, 10 mM Ca in treatment solution or pretreated with 1 and re-equilibrated with 10 mM Ca.

To evaluate the binding capacity and kinetics in bacteria previously exposed to calcium (simulating clinical conditions of enrichment of dental biofilm with calcium, such as after a calcium rinse [10,22]), pellets pretreated with 1 mM Ca were treated with 10 mM Ca. The amount of calcium bound by the 10 mM treatment was the same, regardless of whether the bacterial pellet was pre-reated or not with 1 mM Ca (Fig 2).

Table 2 shows the quantitative and qualitative parameters calculated from kinetics binding curves. The equilibrium binding constant (K_d_) represents the concentration in which half of the binding sites are occupied by calcium, respectively. A low Kd indicates a greater binding affinity. The maximum binding capacity (Ca_bmax_) was higher in those bacteria treated with 10 mM Ca, as compared with those treated with 1 mM Ca; moreover, the equilibrium constant (Kd) was lower in the group treated with 1 mM when compared with 10 mM Ca.

**Table 2:** Calcium binding maximum capacity (Ca_bmax_) and equilibrium constant (K_d_) calculated from the best-fit values of the binding curve kinetics as a function of Ca concentration in treatment solution (mean ± sd, n = 3).

Treatment	Ca_bmax_ (μmol/g pellet)	K_d_ (mM)
**1 mM Ca**	**9.54** ± 1.85	**0.163**
**10 mM Ca**	**32.88** ± 5.93	**3.763**

Kd: concentration at which calcium occupies half of the binding sites.

### Release kinetics

These experiments were done after a 10-min pretreatment (enough time to allow maximum calcium binding according to the previous binding experiment) of the pellets with calcium at 1 or 10 mM.

The equilibrium binding constant (K_d_) and the half-life represents the concentration and time in which half of the binding sites are occupied by calcium, respectively. A low Kd indicates a greater binding affinity.

**Fig 3:**
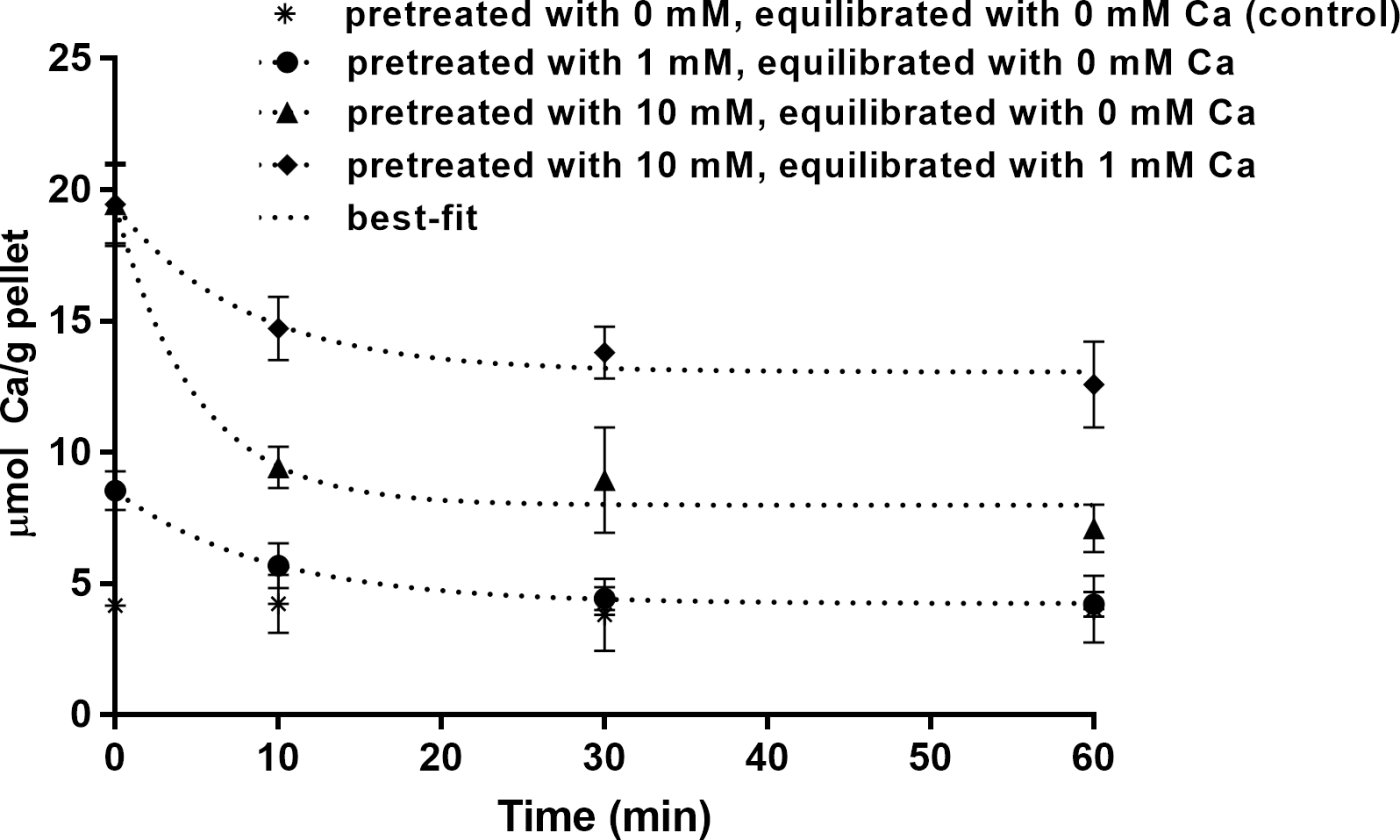
Kinetics of calcium release from *S*. *mutans*. Calcium concentration remaining in the bacterial pellets pretreated with 1 or 10 mM Ca as a function of time of exposure to a calcium-free (0 mM Ca) or a 1 mM Ca solution (mean ± SD, n = 3).

Bacteria pretreated with 1 mM Ca released all bound calcium returning to baseline concentration found in control group. For bacteria pretreated with 10 mM Ca, although at least 70% of release occurred within the first 10 min, it was not completed after 60 min (Fig 3). This incomplete release is confirmed by the estimated calcium bound at the plateau (Table 3).

**Table 3:** Calcium concentration in the bacterial pellet (Ca_b0_) after pretreatment for 10 min with solutions containing 1 or 10 mM Ca, estimated binding at infinity (Ca_b∞_) after equilibrium with calcium-free or 1 mM Ca solution and and half-life values calculated from kinetics release curve, for the different conditions tested (mean ± sd, n = 3).

Pretreatment	Ca_b0_ (μmol/g pellet)	Ca release treatment	Ca _b∞_ (μmol/g pellet)	Half-life (min)
**1 mM Ca**	8.54 ± 0.26	0 mM Ca	4.24 ± 0.22	6.43
**10 mM Ca**	19.44 ± 0.64	0 mM Ca	7.99 ± 0.46	3.40
**10 mM Ca**	19.41 ± 0.58	1 mM Ca	13.06 ± 0.46	5.49

Because the oral fluids will never be depleted of calcium, we tested the calcium release to a 1 mM Ca solution. More calcium remained in the group pretreated with 10 mM Ca and subsequently equilibrated with 1 mM Ca, when compared with the calcium-free solution, a result that is confirmed by the shorter half-life calculated for the latter (Table 3). The half-life values obtained here are estimations based on the protocol used and should be used only to compare the treatments tested here; these values may be lower, but the experimental steps used did not allow us to measure calcium release at shorter periods of time.

### Release as a function of pH

In order to determine the capacity of bacterial-bound calcium to enrich dental biofilm fluid during a pH drop, we treated bacterial pellets previously exposed to Ca to treatments with the same Ca concentration at pHs 5.0 and 1.86, at 37°C. The amount of treatment was reduced to 30% of the pellets weight (similar to the proportion of fluid/solids in *in vivo* plaque [17]). The exposure lasted 10 min (time to reach the lowest pH after dental biofilm is exposed to a cariogenic challenge [23]).

**Fig 4:**
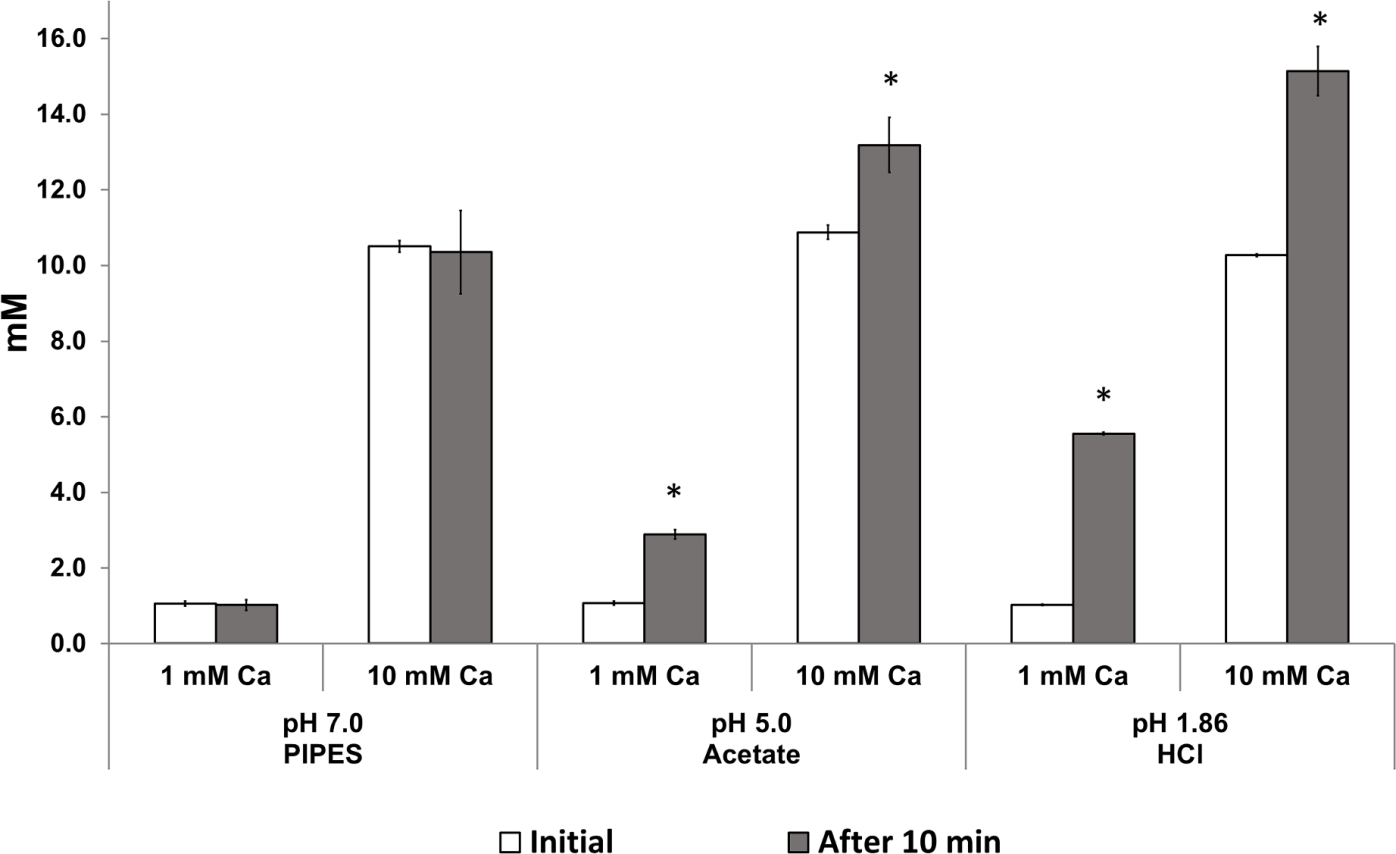
Calcium release from *S*. *mutans* at decreasing pHs. C concentration (mM, mean ± sd, n = 3), in treatment solution initially and after 10 min of equilibrium of bacteria with PIPES buffer pH 7.0, acetate buffer pH 5.0 and HCl pH 1.86 at proportion of 30% the treatment solution per bacterial weight (simulating the liquid-solid proportion in dental biofilms). Pretreatment conditions were 1 or 10 mM Ca. *Initial and final values differ statistically (paired t-test; p<0.05).

Significant increases (p<0.05) in calcium concentration in the test solution were found at pH 5.0 and 1.86 when compared with baseline values, for both pretreatment concentrations (Fig 4). Moreover, the amount of calcium released at each pH was similar (p>0.05 irrespective of the pretreatment with 1 or 10 mM Ca (respectively: pH 5.0 = 0.55±0.03 and 0.69±0.16 μmol Ca/g; pH 1.86 = 1.36±0.01 and 1.46±0.18).

## Discussion

The importance of the calcium bacterial reservoir relies on the fact that Ca^2+^, being one of the ions of hydroxyapatite, may reduce the driving force for tooth demineralization occurring during a pH drop in dental biofilm, when it is released from the bacterial reservoirs (being dislodged from the bacterial anionic sites by the H^+^).

In this study, we validated a method to assess the calcium binding to bacterial surfaces while avoiding, by using an excess volume to bacteria ratio, a change in the calcium concentration in the surrounding fluid, as occurs in the mouth by exposure of dental biofilm to continuously flowing saliva. This change was possible due to the method used to measure bound calcium, i.e., directly from the bacterial pellet, which was shown to be reliable (protocol validation). Although in previous kinetics experiments [12] the treatment concentration was allowed to change, our results confirm that the binding kinetics is fast.

The theoretical equilibrium constant calculated here for both treatment concentrations is of the order of 10^−3^ M. These values agree with dissociation constants typical of phosphates and carboxylic anionic groups (main binding sites in streptococci [5,13]) and with experiments testing the calcium binding to dental bacteria or cell walls [13,24]. Comparing the Kd of the two conditions tested (1 and 10 mM), it can be seen that the calcium concentration that leads to half saturation of binding sites is proportionally lower for the 1 mM group when compared to the 10 mM group, indicating a decrease in affinity by increasing the calcium concentration. Dissociation constants below 2 mM indicate that binding involves two anionic groups on the same cell or two adjacent cells [24], and therefore the results indicate a change in predominance of divalent to monovalent binding between groups with the 10-fold increase in calcium concentration. These results contradict those suggested by Rose et al [7], who inferred that calcium binds predominantly to two binding sites, irrespective of the concentration, as a positive cooperativity, and that only the presence of a monovalent anion, such as fluoride, is able to break the bivalent calcium binding, making sites available for more calcium binding. However, the calcium and fluoride concentrations used by Rose et al [7] favored the precipitation of CaF_2_ minerals, which may have affected the estimation of the calcium binding mechanism in the presence of fluoride.

Our results of binding kinetics also showed that dental biofilm bacteria under equilibrium with calcium concentrations found in saliva/biofilm fluid (around 1 mM) would be able to take up additional calcium when the surrounding concentration increases. In fact, in situ [25,26] and in vivo studies [10] demonstrated that a prerinse of 1 min with solutions containing calcium lactate are able to enrich the biofilm with calcium; such calcium pretreatment may be necessary to boost fluoride retention in dental biofilm (by the formation of calcium fluoride), with positive effects on the reduction of dental demineralization [11].

The slower kinetics of calcium release observed here agrees with the results of Rose et al. [5], who also observed differences in calcium binding and release curves. The explanation for the slower calcium release, when compared with the binding, is sustained by and reinforces the binding mechanism discussed above: if the principal binding at 1 mM Ca is bidentate, a higher degree of affinity between ligand-receptor is expected and a longer time would be necessary to break half of this complex when compared with the monovalent binding predominant in the 10 mM Ca group (Table 2). Regarding the two groups pretreated with 10 mM Ca, the lower value of the dissociation constant in the group re-equilibrated with 1 mM Ca when compared to the calcium-free group could be explained by calcium ions present in the solution, which tend to remake the ligand-receptor complex, decreasing the release rate (Table 3).

The potential of bound calcium to be released to the biofilm fluid and increase the concentration of the ion was estimated in the present study. Rose et al. [5,7] calculated that an increase of approximately 44.0 mM in Ca concentration in the biofilm fluid would happen when the pH is decreased from 7.0 to 5.0 (e.g. during the pH drop induced after exposure of the biofilm to fermentable sugars). However, such high values were never observed in the fluid of dental biofilm in vivo or in situ [4,6,11,19]. Here we observed an increase of approximately 2.0 mM in the surrounding fluid treated with a solution at pH 5.0 (Fig 4), which agrees reasonably with these mentioned studies. The differences in the results observed here and by Rose et al. [5] may be explained by the fact that the latter authors calculated calcium release by the difference in binding capacity at the different pHs, and not by directly measuring calcium release, as attempted here.

The lack of difference in the amount of calcium released from the bacterial pellets treated with 1 or 10 mM Ca (Fig 4) could be explained by two hypotheses: 1. The intensity of the pH drop may not have been sufficient to release ions bound to groups with high affinity to calcium ions, as the phosphate groups of lipoteichoic acid (main Ca-binding sites on *S*. *mutans* [13]), with a dissociation constant (pKa) of 2.1 [27]); or 2. the closed model system and the small volume used limited the ions release to treatment solution. Dental biofilm *in vivo* is bathed in continuously flowing saliva, which dilutes ions released into the biofilm fluid, and a closed model system may not be best strategy to assess this release. Nevertheless, the fluid of the biofilm (estimated here by the 30% volume of treatment solution with respect to the pellet mass) has been considered as an isolated entity between the plaque mass and saliva [28,29,30], due to biofilm diffusion restrictions [31,32,33]. Therefore, the results of release at decreasing pHs can be consider valid for the first minutes during a pH drop in dental biofilm, when the clearance effect of saliva is starting.

In the present study, a 5 to 10 min delay in all times tested in the binding and release experiments occurred, due to the time required to centrifuge and eliminate the treatment solution from the samples. Although this may be regarded as a limitation of the experimental model used, Tatevossian [12] estimated the calcium binding saturation to be reached at 10 min. Therefore, this limitation does not seem to have affected the estimation of a fast calcium binding in the present study, and more importantly, the study on the mechanisms of calcium binding as a function of calcium concentration in the surrounding fluids. The use of a single streptococcal strain also does not seem to impair the conclusions, as the capacity to bind cations is non-specific and varies little among the prevalent streptococcal species in dental biofilm [5].

## Conclusions

Together, our data show that calcium bound to dental biofilm bacteria surface increases under high calcium treatments, is retained for long periods of time, and would promptly be released during a pH drop. Given the importance of calcium as one of the ions of the tooth mineral, this kinetics of binding and release may affect the dynamics of the caries process.
